# Lymphopenia in COVID-19: γδ T Cells-Based Therapeutic Opportunities

**DOI:** 10.3390/vaccines9060562

**Published:** 2021-05-28

**Authors:** Elena Lo Presti, Francesco Dieli, Serena Meraviglia

**Affiliations:** 1 National Research Council (CNR)—Institute for Biomedical Research and Innovation (IRIB), 90146 Palermo, Italy; 2 Central Laboratory of Advanced Diagnosis and Biomedical Research (CLADIBIOR), University of Palermo, 90127 Palermo, Italy; francesco.dieli@unipa.it (F.D.); serena.meraviglia@unipa.it (S.M.); 3 Department of Biomedicine, Neurosciences and Advanced Diagnosis, University of Palermo, 90133 Palermo, Italy

**Keywords:** SARS-CoV-2, gamma delta T cells, COVID-19

## Abstract

Severe acute respiratory syndrome coronavirus 2 (SARS-CoV-2) infection dysregulates the immune system by lymphopenia of B cells, monocytes, eosinophils, basophils, and cytotoxic cells such as CD8, γδ T cells, and natural killer (NK) cells. Despite many studies being conducted to better understand the effects of SARS-CoV-2 on the immune system, many mechanisms still remain unclear, hindering the development of novel therapeutic approaches and strategies to improve the host’s immune defense. This mini-review summarizes the findings on the role of γδ T cells in coronavirus disease 2019 (COVID-19), providing an overview of the excellent anti-viral therapeutic potential of γδ T cells, that had not yet been exploited in depth.

## 1. Introduction

Severe acute respiratory syndrome coronavirus 2 (SARS-CoV-2), a new strain of coronavirus, was named coronavirus disease 2019 (COVID-19) by the World Health Organization (WHO), and it was declared the sixth public health emergency of international concern on 30 January 2020. The European Centre for Disease Prevention and the Control Agency of the European Union have reported more than 99 million cases of COVID-19 worldwide, with more than 2 million deaths.

Asymptomatic subjects, who are fully capable of spreading the virus [1], and SARS-CoV-2 variants detected through viral genomic sequencing remain the main problems. The dramatic emergency has required the development of vaccines in a record time, but more time will be necessary to achieve herd immunity. In the meantime, it is necessary to devise new strategies to efficiently prevent the progression of the disease toward acute respiratory distress syndrome (ARDS), the predominant cause of death in SARS-CoV-2-infected patients.

Most patients with severe COVID-19 have significantly elevated serum levels of pro-inflammatory cytokines, including interleukin (IL)-6 and IL-1β, as well as IL-2, IL-8, IL-17, granulocytes growth factors, interferon-γ-inducible protein 10 (IP10), monocyte chemoattractant protein-1 (MCP1), macrophage inflammatory protein 1 alpha (MIP1α), and tumor necrosis factor alpha (TNFα). Many of them are described as “cytokine storm”, which is comparable but not clinically equivalent to the cytokine release syndrome (CRS) detected in patients receiving treatment with chimeric antigen receptor-transduced T cells (CAR-T) [2]. Cytokine storm is mainly responsible for SARS-CoV-2 infection-mediated toxicity and end-organ damage [3,4]. In addition, several studies have characterized the host–pathogen relationship [5], including immunoprotection and immune dysregulation. SARS-CoV-2 infection induces unregulated innate inflammatory responses and compromised adaptive immune responses, resulting in harmful tissues both locally and systemically [6].

On the basis of the evidence currently available, in patients with severe COVID-19 but not in patients with moderate disease, lymphopenia is a common characteristic, with severely reduced numbers of B and T cells, natural killer (NK) cells, and γδ T cells [5,7,8], as well as a reduced percentage of granulocytes. These characteristics suggest that the modified immune system plays a key role in determining the progression of the disease [9].

Several strategies have been applied, such as antibodies for passive protection, immunomodulatory anti-SARS drugs, and antibodies neutralizing the specific target, such as IL6, IL1, and IL17 [6]. In addition, given the involvement of complement activation, complement inhibitors can reduce inflammatory damage at early stages of the infection [10]. These therapeutic strategies have not proved effective for all patients but have allowed for the management of patients with severe disease at a time when there were no other therapeutic tools.

γδ T cells are among the cells decreased in the lymphopenia state in symptomatic COVID-19 patients [11,12,13,14]. We support the anti-viral potential of human γδ T cells toward virus-infected cells for several reasons: (1) γδ T cells show cytotoxic activity against virus infected cells; (2) they are easy to obtain from blood and culture; (3) they are an unrestricted major histocompatibility complex (MHC); (4) they do not need co-stimulation; and (5) they recognize antigens shared by a number of stressed, tumor, and infected cells, allowing a single γδ T cell to attack a wide spectrum of infected cells. In the following, we describe the specific characteristics of γδ T cells to protect against virus infection and their prospective use in the early phase of SARS-CoV-2 infection.

## 2. γδ T Cells in the Anti-Viral Host Immune Response

A small proportion (1–5%) of circulating CD3^+^ T lymphocytes consists of T lymphocytes expressing the γδ T cell receptor (TCR). Circulating γδ T lymphocytes prevalently express Vγ9Vδ2-encoded TCR, whereas Vδ1 T lymphocytes are resident in the colon epithelia, lung, and intestine [15]. Human Vδ3 T cells compose the majority of non-Vδ1 and non-Vγ9Vδ2 γδ T cells, and are abundant in the liver [16] and in patients with cytomegalovirus (CMV) infection [17] and B cell leukemia [18]. In the absence of antigen processing and MHC restriction, human Vγ9Vδ2 T cells recognize phosphoantigens (PAgs), which are intermediates of the mevalonate pathway [19]. Concentrations of PAgs necessary for the activation of Vγ9Vδ2 T cells are not achieved under physiological conditions but only after infection or tumor transformation [20]. Therefore, from this viewpoint, Vγ9Vδ2 TCR acts in a similar manner to a pattern-recognition receptor (PRR), which detects the metabolic changes observed in transformed or infected cells.

By means of a Vδ2-specific mAb, it has been possible to recognize another subset of γδ T called Vγ9 neg Vδ2^+^ T cells. The latter clonally expand in response to CMV, and, similarly to Vδ1^+^ T cells, the range of pathogens to which they respond is still unclear. Based on Davey’s work [21], the Vγ9 neg Vδ2^+^ T cell responses could be beneficial when adaptive immunity is suppressed in several clinical scenarios, including lymphopenia induced by COVID-19.

Vδ1 T cells express natural-killer group 2, member D (NKG2D), but it is still unknown if they recognize MHC class I chain-related A and B (MICA and MICB, respectively) as well. Vδ1 T cells can also be activated when natural cytotoxicity receptors (such as NCRs, NKp30, and NKp44) bind undefined ligands [15,22]. Vδ3 T cell ligands are not thoroughly defined, and only one study [23] suggested that these cells can be activated by CD1d bound to a glycolipid that has not yet been identified.

Briefly, in this section, we list several studies in which γδ T cells were found to play a protective role in a variety of viral and bacterial infections.

Poccia et al. [24] studied the T cell repertoire of healthcare workers who survived SARS-CoV infection during the 2003 epidemic, finding that their effector memory Vγ9Vδ2 T cell populations were selectively expanded within 3 months of the initiation of the disease. In these workers, αβ T cell pools did not expand. In addition, in vitro studies have shown that activated Vγ9Vδ2 T cells exhibit interferon (IFN)-γ-dependent anti-SARS-CoV activity and are able to directly kill SARS-CoV-infected target cells. These results are consistent with the likelihood of Vγ9Vδ2 T cells playing a defensive function during SARS infection [24].

The role of NKG2D ligands in the identification and killing of CoV-infected cells was demonstrated by Dandekar et al. [25]. NKG2D is an activating C-type lectin NK cell receptor accepted as a potent co-stimulator of human Vγ9Vδ2 T cell cytotoxic functions [26]. Therefore, the authors did not exclude the hypothesis that the detection and killing of SARS-CoV-infected cells may include NKG2D ligands. There are consistent data supporting the idea that Vγ9Vδ2 T cells contribute to promoting antigen processing and presentation, thus providing costimulatory signals to dendritic and αβ T cells [27,28]. Therefore, γδ T cells may also participate in the induction of conventional immune subsets (TCR αβ T cells) against SARS-CoV infection.

In HIV infection, instead, there are significant alterations in the TCR γδ repertoire compared with healthy individuals. These variations are due to the selective conservation (and usually modest expansion) of Vδ1-expressing cells and the concomitant depletion of Vδ2-expressing cells [29]. Recently, the anti-viral functions of human γδ T cells toward influenza viruses have been deeply analyzed, and influenza-reactive γδ TCRs have been defined in the context of γδ-TCRs across the human lifespan. The authors demonstrated that γδ T cells display polycytotoxic and produce IFN-γ in influenza-infected patients. Importantly, the human TCR γδ repertoire seems to be shaped by age, tissue compartmentalization, and the individual’s history of infection, and Vγ9Vδ2 T cells are directly involved in influenza-mediated immunity [30].

The cytotoxic activity of infiltrating γδ T cells in the lungs may even be significant in the early stages of *Mycobacterium tuberculosis* infection by restricting the quantity of bacilli and favoring the development of the protective αβ immune response. γδ T cells provide a crucial early IFN-γ burst that conditions dendritic cells (DCs) for the effective priming of CD8 T cells and the complete development of a protective response [31,32]. This protective role has also been demonstrated in CMV infection and Herpesvirus infection [33,34].

The proteomic analysis of 101 plasma proteins of 40 hospitalized COVID-19 patients who did or did not require Intensive Care Unit (ICU) admission revealed that the sustained variations found over the disease course were in Caspase 8 (CASP8), TNF Superfamily Member 14 (TNFSF14), Transforming Growth Factor Beta 1 (TGFB1), and Hepatocyte growth factor (HGF), which were associated with apoptotic pathways and inflammation. Therefore, this finding could be considered to underpin T cell apoptosis as a fundamental feature that leads to SARS-CoV-2-specific immune pathology [35]. Thus, in the following section, we discuss the recent knowledge acquired on γδ T cells.

## 3. Lymphopenia and γδ T Cell Exhaustion in COVID-19

Lymphopenia, or lymphocytopenia, is the condition generated by a low number of lymphocytes in the blood. Although T cells could be initially increased at the onset of COVID-19 infection, during the progression to severe pneumonia, patients appear to have low number of lymphocytes, which display activation or exhaustion/senescence markers as well as an altered expression of master regulators and multiple chemokine receptors [36]. In addition, the overall number of adaptive T cells decreases dramatically, especially in elderly patients over 60 years of age and in those needing intensive care [37]. Studying an immune signature that characterizes SARS-CoV-2 infection could offer many potential opportunities for immunotherapy and increase the knowledge of the underlying mechanisms of the development of the illness.

Laing et al. [14] tried to find a common COVID-19 immune signature from 63 hospital-treated patients (Table 1). In an effort to characterize the host–pathogen relationship, they also considered sex, ethnicity, age, underlying illness, and clinical presentation. Interestingly, the authors noted that there was a simultaneous mechanism of suppression and activation of T lymphocytes. In particular, they observed how CD8^+^ cells and γδ T cells were most clearly affected than CD4 T and NK cells. Moreover, severe losses of Vγ9Vδ2 cells in the blood substantially shifted the compartment composition toward Vδ1^+^ cells. Thus, in particular, the depletion of Vδ2^+^ T cells or other immune cells such as plasmacytoid Dendritic Cells (pDC) and basophils could provide the means to monitor and better understand the specific components of COVID-19 infection.

A common COVID-19 immunophenotype is represented by CD8+ T cells coexpressing both PD-1 and TIM3 (exhausted-associated markers), but their hyperexpression was observed during the progression of symptomatic stages [37,38]. The T cell dysregulation induced by SARS-CoV-2 infection affects memory T cells, upon which adults are presumably more reliant than children because of thymic involution. Thus, T cell dysregulation might determine a decrease in T-cell-mediated immunoprotection, and the expansion of the pool of naive T cells may explain the relatively reduced severity of COVID-19 infection in children [39].

Knowledge of the mechanism through which SARS-CoV-2 might induce a low level of γδ T cells is still limited. Recently, many authors have evaluated an upregulation of NKG2A expression on NK cells; in COVID-19-infected lung epithelial cells, there is an overexpression of HLAE. NKG2A is a heterodimeric inhibitory receptor that binds to the nonclassical HLA class I molecule (HLA-E) on NK cells [40,41]. Peptide-loaded HLA-E binds to NKG2A, and the latter transduces inhibitory signaling through two tyrosine-based inhibition motifs (ITIMs), which suppress NK cytokine cytotoxicity and secretion [40]. Even more interesting is the decreased NKG2A expression in NK cells of convalescent subjects [42]. Even if the enhanced expression of NKG2A has not been revealed in the γδ T cells of COVID-19 patients, it is known that small intestinal CD8^+^ TCR γδ T cells of celiac patients might express NKG2A in the memory subsets [43,44]. Hence, more studies are needed to verify if SARS-CoV-2 deactivates the antiviral immunity [45] by activating NKG2A; this may explain, in turn, why γδ T cells are decreased at onset [46].

Immunosenescence is defined as a lack of clonal T cell diversity and a contraction of naïve T cells with proliferative ability [47]. A more restricted T cell repertoire is likely to be more inclined toward antigen-mediated exhaustion during chronic viral infections, but it cannot be excluded that multiple pathways can work together to induce lymphopenia [39]. SARS-CoV-2 can target lymphocytes directly or damage lymphoid organs. Because patients with a severe COVID-19 phenotype have increased levels of lactic acid in the blood, lymphopenia may be related to such metabolic molecules [48].

In a study of the blood and airways of patients with severe COVID-19 infections, Jouan et al. [13] analyzed the heterogeneous class of unconventional T lymphocytes such as mucosal-associated invariant T (MAIT) cells, γδ T, and invariant Natural Killer T (iNKT) cells of 30 patients who were admitted to the ICU (Table 1). By focusing on γδ T subsets, distinct changes with a small decrease in Vδ2^+^, a modest rise in Vδ1δ2^−^, and no change in the Vδ1^+^ subset were observed. To monitor local inflammation, they analyzed unconventional CD3^+^ cells in the endotracheal aspirates (ETAs) of COVID-19 patients. MAIT and γδ T cells were detected in the ETA of only 12 patients, whereas unconventional T cells were not detected in the ETA from non-COVID-19 patients.

**Figure 1:**
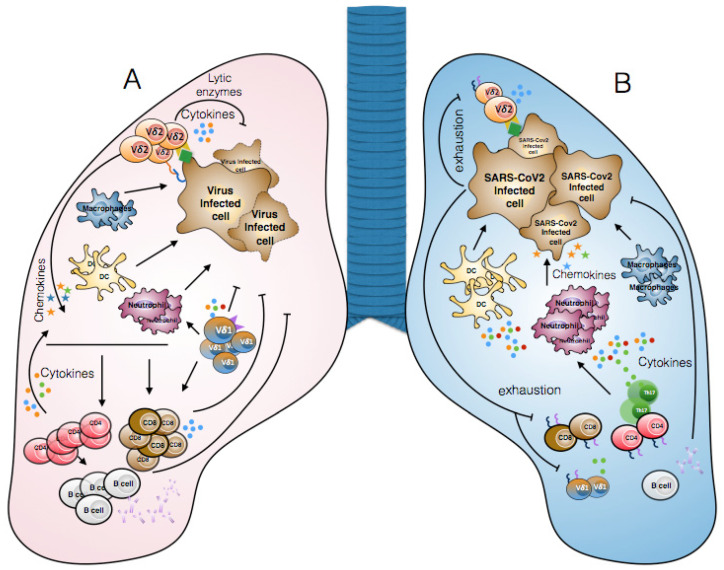
Immune response during common viral infections and SARS-CoV-2 infection. (**A**) γδ T cells directly recognize virus-infected cells through the TCR, NKG2D, or CD16 and kill them via lytic enzymes (perforin, granzyme) or TRAIL-mediated pathways. γδ T cells indirectly participate in anti-viral immune responses by producing cytokines (for example, IFN-γ) which promote the activation of M1-type macrophages, CD4, and CD8 T lymphocytes. Moreover, γδ T cells can produce IL-17, contributing to the recruitment of pro-inflammatory cells such as neutrophils. (**B**) In severe cases of SARS-CoV-2 infection, the recruitment and activation of neutrophils, monocytes/macrophages, and dendritic cells results in the production of various pro-inflammatory cytokines which cause the so-called “cytokine storm”. Another consequence of SARS-CoV-2 infection is lymphopenia (NK, B, CD8, CD4, and γδ T cells) and lymphocyte dysfunction due to the expression of exhaustion markers (for example, PD1 and TIM3). Finally, SARS-CoV-2 affects the immune response, generating an inappropriate defense mechanism that permits viral spread.

In addition, the levels of an activation marker such as CD69 on the γδ T cells from COVID-19 patients were not substantially altered relative to non-COVID-19-infected controls, suggesting that this activation status could be a general reflection of a severe condition rather than a SARS-CoV-2 signature. Regarding cytokine-producing γδ T cells, compared with healthy subjects, less IFN-γ and more IL-17A (although the levels detected were always low) were released by γδ T cells from COVID-19 patients [49] (Figure 1). This finding coincides with the data reported by Cossarizza et al. on a total of 39 patients presenting an altered differentiation in various T cell subtypes with a skewing toward Th17 [38].

Carissimo et al. [12] identified immature neutrophils, CD8 T cells, and Vδ2 T cells in a cohort of 54 COVID-19 patients. They also analyzed CD38 expression, which is thought to play a role in the defense against infections. They found that all CD8^+^ T cell differentiation stages Vδ1 and Vδ2, except for Vδ2 TEMRA, had higher CD38 expression with a slightly increased Vδ1 T cell subpopulation. CD4^+^ T cells, conversely, did not display any difference in the expression of the CD38 activation marker. As a consequence, CD8 T cells and Vδ2 T cells have to be considered as key immune cell populations opposing COVID-19 infection [16]. Across the clinical severity spectrum, these cytotoxic cells undergo major changes in cell counts. In reality, their numbers would have a precocious and robust prognostic value. A rise in the number of neutrophils and the neutrophil-to-lymphocyte ratio typically suggests a greater severity of disease and a worse clinical outcome [7]. There was a highly increased neutrophil-to-lymphocyte ratio in many, but not all, patients [45].

However, Vδ1 and Vδ2 T cells do not seem to increase in absolute numbers in recovered subjects, and their cytotoxic function could be modified by several metabolites. In support of this, several studies have shown a lipidomic alteration in the plasma of cured COVID-19 patients [50,51], which could be indirectly connected with the failure to restore the effector function of γδ T cells [52]. Indeed, the increase of triglycerides and decrease of cholesteryl esters (ChE) in cured COVID-19 patients could justify the decreased frequency of Vγ9Vδ2 T cells. In fact, lipid metabolites can modulate the phosphoantigen-mediated Vγ9Vδ2 T-cell function, impairing IFN-γ production and reducing cytotoxic activity [53].

## 4. Contribution of γδ T Cells to Anti-SARS-CoV-2 Therapy

The main goal of immunotherapy is to produce a long-lasting, successful immune response, particularly mediated by cytotoxic CD8 T cells but also by CD4 T cells [54]; however, despite all efforts, durable responses are rarely reached. Unlike CD4 or CD8 T cells, the special characteristics of γδ T cells make them strong candidates for immunotherapy. Many studies in the oncology field have enabled the creation of new therapies based on the use of γδ T cells [55,56].

The results of γδ T-cells-based clinical trials have been extensively discussed in many reviews [15,57] and are based on the following two procedures: (1) zoledronate and IL-2 in vivo administration to safely expand circulating Vγ9Vδ2 T cells in the patient and (2) adoptive transfer of Vγ9Vδ2 T cells expanded ex vivo with zoledronate and IL-2. These procedures could be adapted to reinforce γδ T cells in patients during the early stage of SARS-CoV-2 infection, as therapies based on drugs to modulate the adaptive immune system of COVID-19 patients and to reduce the consequence of acute respiratory syndrome (ARS) are currently not entirely satisfactory [58].

Brufsky et al. [59] hypothesized that the expansion of Vγ9Vδ2 T cells through the administration of amino-bisphosphonates is useful for two further reasons: (i) these molecules can operate on the DCs to further activate the initial immune response to the virus, and (ii) as prenylation inhibitors of small GTPases in the endosomal pathway of DC, these could avoid the expulsion of lysosomes carrying SARS-CoV-2 virions. Polito et al. [60] reported a clinical-grade protocol to isolate and efficiently expand high numbers of polyclonal γδ T cells both manually and automatically. This procedure also provides proof of the possibility of further improving the anti-viral efficacy through gene modification. Through the introduction of the costimulatory molecules CD86, 4-1BBL, CD40L, and the pp65-CMV antigen, engineered artificial antigen-presenting cells (aAPCs) allow for a broad expansion of γδ T cells. This type of expansion maintains the polyclonal phenotype, with a significant representation in the memory Vδ1 subgroup.

Antonioli et al. [42] proposed monalizumab, a first-in-class humanized IgG4 targeting the NKG2A receptor expressed on cytotoxic cells. Monalizumab is able to interrupt the inhibition of NK and CD8 T cells, leading to the return of proper antiviral activity by the accumulation of IFN-γ. As an alternative, other authors proposed an anti-BTN3A antibody (ICT01), which is currently being used in anti-cancer Phase 1 studies [4]. Finally, another strategy could be the use of monoclonal antibodies in precocious infection to protect the cytotoxic γδ T cells from blocking inhibitor receptors to strengthen γδ T cell therapy against COVID-19. We want to highlight that there is renewed interest in studying the interactions of γδ T cells and B cells. It was demonstrated that γδ T cells can favor the murine antibody production by B cells by secreting IL-4 and IL-10 [61]; today, several researchers are investigating the involvement of γδ T cells in post bone marrow B cell maturation and extrafollicular/germinal center B cell differentiation into plasma cells, as previously reviewed [62]. As IgG production is involved in some essential immunotherapy protocols, this aspect, in the SARS-CoV-2 pandemic period, opens a new and broad field for further exploration. If γδ T cells influence B cells in the opposite direction, it was speculated that γδ T cells are subject to influence by the IgG repertoire, which could act as a ligand of γδ TCR [63].

## 5. Future Directions and Concluding Remarks

As vaccines are the best solution to combat the virus, the strategies proposed and the later considerations could offer alternative treatments to restrain the spread of COVID-19. More in-depth studies on the immunophenotyping of γδ T cells should be conducted, as well as on assessing the expression of NKG2A. Targeting NKG2A or using antibodies anti-immune checkpoints could prevent the functional exhaustion of cytotoxic γδ T cells, and consequently contribute to virus killing in the early stage of SARS-CoV-2 infection. More information is needed about the relationship between γδ T cells and B cells to evaluate potential immunotherapy strategies. Finally, it is also necessary to analyze the characteristics of γδ in cured or asymptomatic subjects in order to create specific γδ-based immunotherapies to fight SARS-CoV-2 infection in the early stage of infectious disease.

## Figures and Tables

**Table 1:** Summary of the number of patients and their clinical characteristics in the studies on γδ T cells. Non-COVID-19 patients and healthy subjects have been used as controls.

Ref.	Author	Patients Enrolled	Early Infection	Hospitalized ICU			Died	Non-COVID-19	Healthy Subject
n°8	Rijkers et al.	24	-		18		6	-	_
n°11	Wilk et al.	7			6		1	-	6
n°12	Carissimo et al.	54	-		28		-	-	19
n°13	Jouan et al.	30	-		30		-	17	20
n°46	Lei et al.	38	-		-		_	-	18
		**Patients Enrolled**		**Low**	**Moderate**	**Severe**		**Non-COVID-19**	**Healthy Subject**
n°14	Laing et al.	63	-	6	26	31		42	23

ICU: Intensive Care Unit.
